# Different Synergy in Amyloids and Biologically Active Forms of Proteins

**DOI:** 10.3390/ijms20184436

**Published:** 2019-09-09

**Authors:** Piotr Fabian, Katarzyna Stapor, Mateusz Banach, Magdalena Ptak-Kaczor, Leszek Konieczny, Irena Roterman

**Affiliations:** 1 Institute of Computer Science, Silesian University of Technology, Akademicka 16, 44-100 Gliwice, Poland; 2 Department of Bioinformatics and Telemedicine, Jagiellonian University—Medical College, Łazarza 16, 31-530 Kraków, Poland; 3 Faculty of Physics, Astronomy and Applied Computer Science, Jagiellonian University, Łojasiewicza 11, 30-348 Krakow, Poland; 4 Chair of Medical Biochemistry, Jagiellonian University—Medical College, 31-034 Krakow, Poland

**Keywords:** misfolding, amyloid, secondary structure, α-synuclein, V domain of the immunoglobulin G light chain, force field

## Abstract

Protein structure is the result of the high synergy of all amino acids present in the protein. This synergy is the result of an overall strategy for adapting a specific protein structure. It is a compromise between two trends: The optimization of non-binding interactions and the directing of the folding process by an external force field, whose source is the water environment. The geometric parameters of the structural form of the polypeptide chain in the form of a local radius of curvature that is dependent on the orientation of adjacent peptide bond planes (result of the respective Phi and Psi rotation) allow for a comparative analysis of protein structures. Certain levels of their geometry are the criteria for comparison. In particular, they can be used to assess the differences between the structural form of biologically active proteins and their amyloid forms. On the other hand, the application of the fuzzy oil drop model allows the assessment of the role of amino acids in the construction of tertiary structure through their participation in the construction of a hydrophobic core. The combination of these two models—the geometric structure of the backbone and the determining of the participation in the construction of the tertiary structure that is applied for the comparative analysis of biologically active and amyloid forms—is presented.

## 1. Introduction

The issue of amyloid transformations is a central point of interest for specialists in the field of protein structure analysis [1,2,3,4,5,6,7,8,9,10]. Paradoxically, misfolding proteins will prove helpful in answering the question about proper protein folding [1]. The availability of amyloid structures through the use of the solid-state NMR technique enabled the analysis of the specifics of these structures [11,12]. The amyloid forms of Aβ (1–42) causing Alzheimer disease are present in PDB [13,14,15,16]. The availability of the amyloid form of the tau protein has revealed the possibility of polymorphism of fibril structures [17] or the α-synuclein structure (called as ASyn in this paper), where only a selected fragment of the chain forms the fibril form [18]. The special place of α-synuclein also results from the availability of the structure both in the amyloid fibril [18] and in the biologically active form—micelle-bound α-synuclein [19]. Immunoglobulin V domain is also available in biologically active amyloid structural forms. These structures are available in addition to numerous forms of the Fab fragment in the form of a dimer called Bence-Jones (two lambda light chains) [20] and in the form of amyloid [21]. Especially the availability of the two last mentioned proteins creates the possibility of comparative analysis, which may suggest a potential mechanism of amyloid transformation.

The specificity of globular structures in combination with amyloid structures suggests the presence of different synergies. The structure of proteins is the result of the cooperation of amino acids, which more or less participate in the stabilization of the final product. This stabilization comes from the presence of a more or less ordered construction of the hydrophobic core. This ordering is understood in the fuzzy oil drop model as the striving to generate by ordering the distribution of hydrophobicity in the form of a hydrophobic core [22,23]. The form of the hydrophobic core in the case of globular proteins appears to be in the form of a spherical micelle. Meanwhile, amyloids seem to prefer the form of a ribbon-like micelle, also with a more or less centralized band system with a high concentration of hydrophobicity [24,25]. The formation of the correct and misfolded form of the protein has its source in the conformation of peptide bonds, which can be expressed by means of Phi and Psi angles. It may also be expressed by geometric parameters, such as the size of the local radius of curvature or the angle of aperture between two adjacent planes of peptide bonds [26,27,28,29].

The characteristics of the geometric structure of polypeptides in their naturally folded form and their amyloid forms in conjunction with the functionality of the final forms seems to reveal the different nature of synergy that is present in the construction of polypeptide structures. This approach is the subject of the analysis presented here.

## 2. Results

### 2.1. Comparative Analysis of the ASyn Structure

The structure of the amyloid form ASyn available in the PDB database shows the presence of fibrillar order only in the segment 30–100. That is why this fragment of the whole chain is analyzed here.

#### 2.1.1. Comparative Analysis of the Distribution of Phi and Psi Angles of the Asyn Structure in the Form of Micelle-bound (Single Molecule) and in the Form of Amyloid Fibril

**Figure 1:**
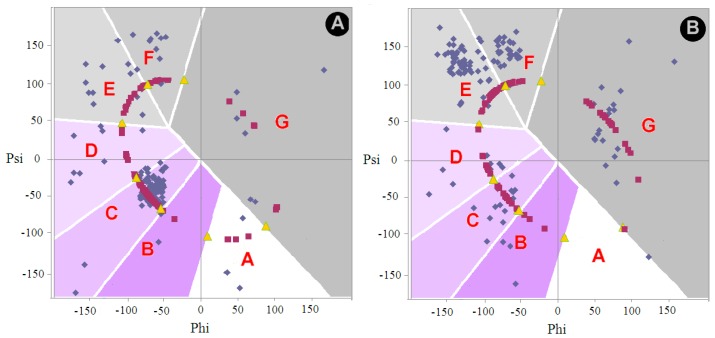
The distribution of angles Phi and Psi on the background for A—micelle-bound ASyn (1XQ8), B—amyloid form (2N0A). The image is limited to the fragment 30–100. Blue points—Phi, Psi angles for structures available in PDB, red points—Phi_e_, Psi_e_, yellow points—zone boundaries for structural codes. Phi_e_ and Psi_e_—position of Phi and Psi angles after transformation to the ellipse (subscript “e”) (shortest distance criterion). This operation is assumed as a step-back of the final structure of protein under consideration to its early structural form. A–G – zones on Ramachandran map distinguished according to local maxima on the ellipse path.

Phi and Psi angles were determined for the ASyn—micelle bound (1XQ8) structure and its amyloid form (2N0A). On their basis, the status was established under the structural codes A–G (Figure 1). The ellipse path was defined as the result of geometry analysis, i.e., possible geometric forms of pentapeptides. Two geometric parameters are: The radius of curvature R, which is the result of the V-angle—dihedral angle between two adjacent peptide bond planes. The angle measure is a simple consequence of the Phi and Psi rotations. The zones A–G marked on the Ramachandran plot represent the separation of the map according to the local maxima of the angles Phi_e_ and Psi_e_ (the subscript “e” denotes the belonging to ellipse path) along the ellipse after projecting the point (Phi, Psi) on the ellipse (shortest distance criterion). Maxima were determined based on a non-redundant set of proteins (see Section 3 Materials and Methods). The ellipse path was treated as a limited conformational subspace.

The status statement of residues reveals those positions that have changed as a result of amyloid transformation (Table 1).

The distribution of Phi and Psi angles for both discussed forms for the segment 30–100 is illustrated in Figure 1. The current division into zones associated with a given local maximum reveals the dominant location of the Phi and Psi angles in the helix area. However the distribution of angles in fibril indicates a dominant presence in the E region with a negligible contribution of the conformation for the F code. This proportion suggests the presence of long fragments with a β-structure with a few residual conformation termed F. Status E means the advantage of almost straight forms, while F means the appearance of a twist terminating β-propagation. Analysis of the location of the Phi and Psi angles in relation to the geometry represented by the maps (see Section 3 Materials and Methods for details) suggests the presence of segments with high values of the angle V (close to 180 deg) and a large value of the radius of curvature (over 8 on the natural logarithm scale). The location of the Phi and Psi angles in the case of the amyloid ASyn form indicates the predominant presence of segments in a form close to a straight line.

Comparison of the status of Phi and Psi angles (within structural codes) (Figure 1) illustrates the flow of angles from the mainly C (helical) zone, which is quantified in Table 1.

These two proteins are an example of a radical change from a helical form with a low radius of curvature to a form containing the opposite form with an extremely large radius of curvature.

#### 2.1.2. Determining the Status of Individual Residues in Relation to the Structure of the Hydrophobic Core

**Figure 2:**
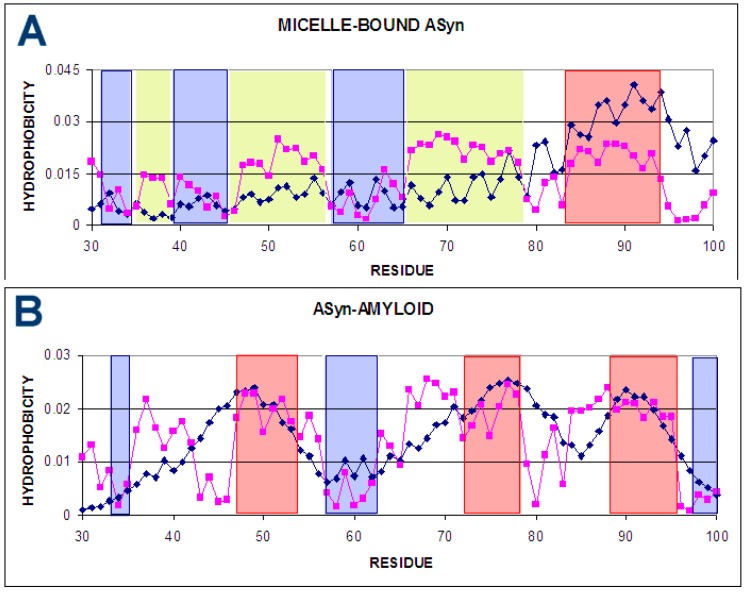
T and O distribution for: (**A**) micelle-bound ASyn (1XQ8); (**B**)—status of the chain C as part of the entire fibril (all chains are part of the protein for which 3D Gauss function is generated) (2N0A); the charts represent distributions for the 30–100 segment for all discussed forms. The positions (sections) involved in the construction of the hydrophobic nucleus are marked red. The blue positions—accordant status with low (surface) hydrophobicity level. The yellow segments distinguish the fragments representing evident discordance between expected and observed hydrophobicity level. These fragments are distinguished in 3D presentation in Figure 3.

The comparison of T and O hydrophobicity distributions for both discussed structural forms reveals the participation of individual residues in the structure of the hydrophobic core (Figure 2). The differentiation of the role of individual residues seems to be obvious.

The status of segment 30–100 in micelle-bound ASyn is described by parameter RD = 0.672. The comparison of the distribution of T and O shows significant differences between them (Figure 2A). A weak mapping of the hydrophobic nucleus is visible, which is expected mainly for positions 80–100. In the 30–80 segment, significant local hydrophobicity surpluses are observed. Taking into account the fuzzy oil drop model, such excess of hydrophobicity suggests hydrophobic exposure on the surface of the molecule. This situation is associated with the possibility of interaction with analogous proteins with a local excess of hydrophobicity exposed on the surface. In this particular case, it can be speculated that these areas are probably involved in interaction with the micelle, with which this protein is complexed in an experiment that allows the structure of this protein to be determined [18].

The yellow line in Figure 2A indicates residues potentially involved in interaction with the micelle as representing local excess of the hydrophobicity.

A completely different distribution of hydrophobicity for segment 30–100 is observed for the amyloid form ASyn (Figure 2B). Local maxima are located in similar sections of the chain to some extent (accordant fragments are nearby positions 30 and 60 and discordant are nearby position 90). However, the relationship between the status of these sections and the expected distribution is completely different. This is due to the different distribution of T, where a much smaller number of local maxima is expected. Their shape is more continuous and uniform compared to the jagged distribution observed in the micelle-bound. The status of the discussed fragment 30–100 in the fibril structure (all chains present in generating the 3D Gauss function) is expressed by the value of RD = 0.531 and for the individual chain RD = 0.683 (3D Gauss function generated for a single chain). This means that none of the forms meets the condition of building an ordered hydrophobic nucleus.

The change of role of particular residuals towards the expectation of a system treated as a synergy effect is shown in Table 2, Table 3 and Table 4.

The status of the residue identified as not-accordant was determined by stepwise elimination of those residues whose exclusion causes the decrease of the RD value below 0.5. Residues that make up the nucleus were determined on the basis of high values of both T and O. Compliance status refers to residues showing O and T compliance for low values of hydrophobicity O and T.

In Table 2, there is an increase in the number of residues involved in the structure of the hydrophobic nucleus in fibril (from 11 to 25). A similar situation occurs in the analysis of the relationship of status of a single fibrillar chain to the status shown in micelle-bound ASyn (Table 3). In Table 4, the presence of the highest numbers on diagonals—similar status of residues in a single chain as in fibril—is noteworthy. Non-diagonal numbers result from a different form of 3D Gauss function for the entire fibril, compared to the form of 3D Gauss function for a single chain.

The analysis of the results given in Table 2, Table 3 and Table 4 shows the change in the synergy system in the structure of the system in the discussed forms of protein structures. The comparison of these values with the results of Table 1 indicates a change in the structural form that accompanies the change in the status of individual residues in the construction of the synergistic form of the discussed structures.

The changes given in Table 1 and Table 2, Table 3 and Table 4 are coupled together. The changes shown in Table 1 accompany the those shown in Table 2, Table 3 and Table 4. The open question about the cause-and-effect relationship remains: Does the difference in the logic of the hydrophobic nucleus structure generate other values of Phi and Psi angles, or is this relationship inverse?

**Figure 3:**
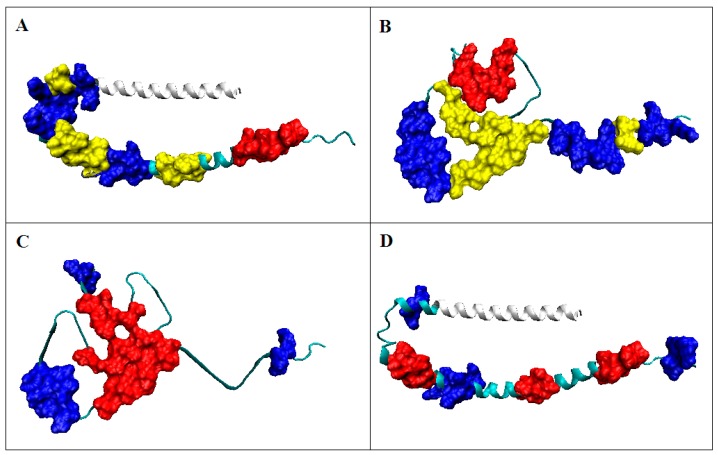
3D structure presentation; (**A**) 30–100 in form of micelle-bound (profile on Figure 2A); (**B**) Asyn in fibrilar form—residue distribution according to the criterion resulting from the structure micelle-bound (Figure 2A.); (**C**) Asyn in fibrilar form—residues distinguished according to the distribution shown in Figure 2B; C—Asyn micelle-bound with residues distinguished according to profiles as shown in Figure 2B.; Marked: Red residues—hydrophobic core, blue—low values according to the model; white fragment—segment 1–30 not included in amyloid fibril. The yellow fragments visualize the position of those distinguished in Figure 2 as a significantly discordant level of observed hydrophobicity in respect to the expected one.

In the case of ASyn—considering the micelle-bound form—the structure was subordinated to the interaction with micelle. Hence, local hydrophobicity maxima are observed in the profile in places where low levels are expected. Residues involved in interaction with micelles loose this interaction in the aquatic environment. They generate a structure that is the result of the action of an external force field which is the aquatic environment. The impact of this environment is expressed in the orientation of the folding process towards the generation of a hydrophobic core. This process runs regardless of its larger or smaller reproduction to the distribution expected by the 3D Gauss function (Figure 3). Clearly, an ASyn sequence chain cannot generate its own globular form with an ordered nucleus similar to the Aβ (1–42) [30,31] and tau [32] amyloid proteins.

It is obvious that not all residues represent a status consistent with the theoretical distribution. It can be speculated that the residues that make up the hydrophobic core play the role of a driver. The residues representing a status inconsistent with the expected distribution can be described as passengers.

However, it is clearly seen that the residues forming the hydrophobic core accept different cooperativity. Other residues in the chain simultaneously get exposed toward the water environment.

The conclusion based on the structure of fibril form is that the main “driver” is the generation of the hydrophobic core (together with the exposure of hydrophilic residues on the surface of the fibril). The discordant residues seem to be by the “passengers”.

### 2.2. Analysis of the V Domain of the Light Chain (lambda) of Immunoglobulin G in its Amyloid Form

Similar to the ASyn analysis, the comparative analysis of two forms of domain V was performed. The domain is present in the form of Bence–Jones protein dimer (4BJL [20]), as well as in the amyloid form available in PDB under the code 6HUD [21].

It should be noted that the sequences in the proteins: Immunoglobulin G light chain V domain and that are present in 6HUD amyloid are only 42.8% compatible. However, the comparison of the intrinsic hydrophobicity distribution in both chains, segment 1–37 shows a correlation coefficient of 0.72, while the correlation coefficient for segment 66–105 reaches the same value of correlation coefficient after eliminating certain residues (67, 81, 83, 88, 91, 92, 94, 98, 102). The listed resides will be discussed later in the analysis. Sequence variability within the V domains of the IgG molecule is highly relative to other proteins in the human body [33]. Therefore, the presence of sequence differences with respect to the sequences of compared proteins is slightly less important than for other proteins. However, the residues listed above will be considered later in this analysis. Moreover, a repeated sandwich structure is present in almost all V domain of immunoglobulins (as well as C domains). This similarity is observed despite numerous sequence differences. This is why the adoption of structural changes in the transition of the sandwich form into the amyloid form may authorize such a transformation of the V domain of immunoglobulin G.

#### 2.2.1. Comparative Analysis of Phi and Psi Angle Distribution of the V Domain of Immunoglobulin G in Native Form (Bence–Jones Protein—Light Chain Dimer) and in the Form of Amyloid Fibril

**Figure 4:**
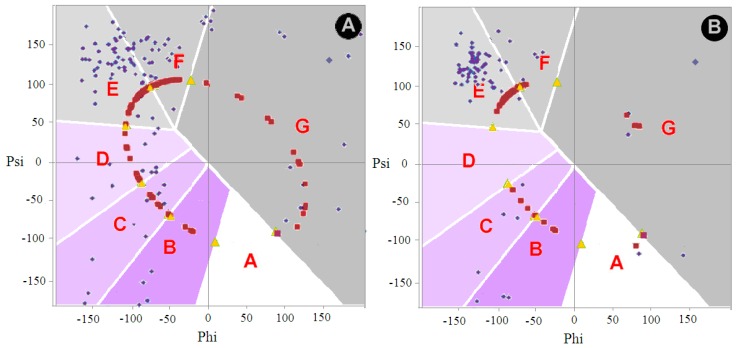
Phi, Psi angle distribution in the structure: (**A**) Immunoglobulin G chain light domain V (4BJL); (**B**) amyloid form (6HUD); only angles for 2–37 and 66–105 are shown in the maps. A–G—zones on Ramachandran map distinguished according to local maxima on the ellipse path.

The location of the Phi and Psi angles in the Bence–Jones (physiological however pathological form of the dimer) and amyloid form suggests the presence of a β-structural form (characterized by large value of radius of curvature) together with forms characteristic for the F code. The comparison with the angle distribution for the amyloid form shows a reduction in the number of conformation residues ending in β-structural sections (Figure 4).

The distribution also indicates the presence of forms with a maximum radius of curvature with the elimination of conformation corresponding to the conformation identified as F.

Changing the conformation to an E-type structure from the other zones is visualized in Table 5.

The analysis of the maps (Figure 4) and Table 5 indicates a significant increase in the E conformation with a significant reduction in the representation of the F conformation. The number of residues representing the F conformation also results from the change in the structure of C, D, and G. See Section 3. for details. With the set of angles in Figure 4 suggests a significant increase in the presence of straight chain structures with few residues representing a structure with a smaller radius of curvature.

The status determined on the basis of fuzzy oil drop model of the discussed polypeptides is presented in Table 6.

The amino acid status analysis summarized in Table 7 indicates a higher contribution to the structure of the hydrophobic nucleus in the V domain. The diversity shown reveals a change in the role played by a given residue in both examples discussed.

**Figure 5:**
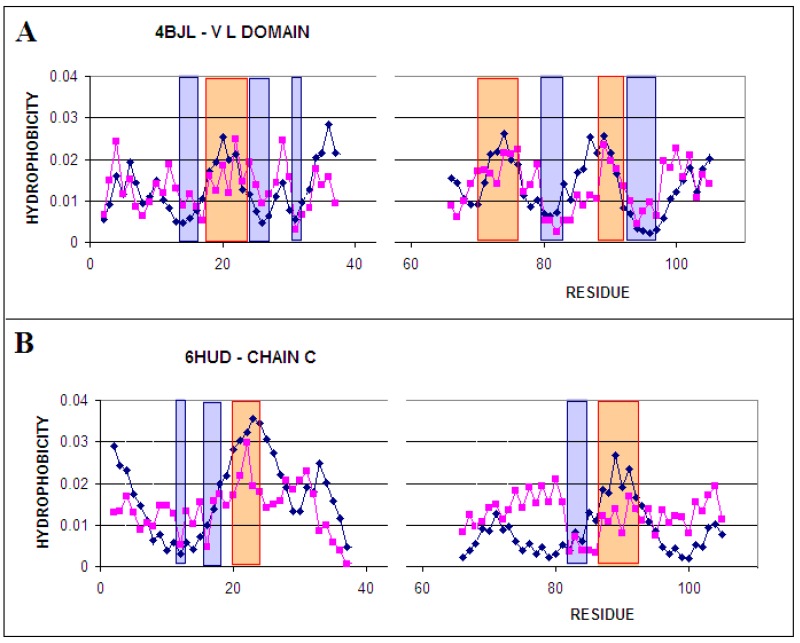
T and O hydrophobicity distribution for: (**A**) Light chain V domains; (**B**) in amyloid form; only segments 2–33 and 66–105 are taken under consideration

**Figure 6:**
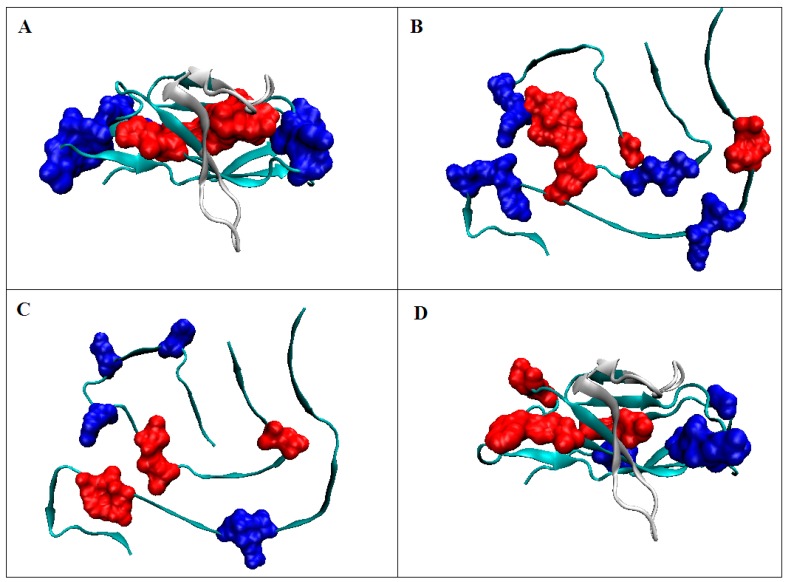
3D presentation of structures: (**A**) IgG light chain V domain with red residues forming the hydrophobic core; blue indicates residues with low hydrophobicity, as expected, located on the surface. Criteria of identification according to Figure 5A; (**B**) amyloid C chain (6HUD) with distinguished residues making up the hydrophobic core of the V domain (as in Figure 5A); (**C**) amyloid C chain (6HUD) with distinguished residues making up the hydrophobic core according to the hydrophobicity distribution in this chain (Figure 5B); (**D**) IgG light chain V domain with residues differentiated according to the profiles shown in Figure 5B. Red—residues belonging to hydrophobic core, blue – residues on surface with low hydrophobicity level—both selections accordant with the fuzzy oil drop model

The location of the residues that make up the hydrophobic core in the V domain indicates its central location of the molecule with the current polar coating exposed on the surface (Figure 5A). Elimination of several residues (they are counted as not-accordant) from the distribution are shown in Figure 6A results in obtaining RD values below 0.5. The distribution of the residues constituting the hydrophobic core in the case of the amyloid form of this chain indicates their high dispersion (Figure 5B). Similarly, the residues that make up the polar outer mantle occupy only a few cases of favorable positions from the point of view of the stability of the hydrophobic nucleus in this structural form.

The distribution of residues constituting the components of the hydrophobic nucleus in the amyloid indicates their central location in fibril. The fragments identified on the profile (Figure 5B) as being part of the polar coating also occupy the appropriate positions. However, significant sections are present in the fibril form which do not meet the criterion of a proper fit.

Residues forming a hydrophobic nucleus in fibril in the structure of the V domain are located in one specific domain area. It covers both the segment with high hydrophobicity and fragments that in the V domain play the role of a polar mantle. This reveals the principle of a fuzzy oil drop model, in which, apart from intrinsic hydrophobicity, the status of a given residue is also influenced by its surroundings. It is assumed that the mechanism of transformation can be recognized by monitoring the structural changes necessary in this case. The structural codes based on the hypothetical model for early stages of polypeptide chain folding are assumed to deliver the possible tool for simulation of the complete folding process. If it becomes possible, the conditioning for such a process may appear recognizable. The link between the geometrical transformation with the specificity of the final form of protein under consideration, which is the effect of the active participation of the water environment, opens up such a possibility.

#### 2.2.2. Participation of the V domain in the Construction of Complexes in the VL-VL (Bence–Jones Dimer) and VL-VH (FAB Fragment of IgG) Fragments

Immunoglobulin V domains in biologically active forms are in the form of V domain dimers derived from the light chain and heavy chain of VL–VH. Under physiological conditions, in a pathological protein called Bence–Jones, the VL domain remains in the form of the VL–VL complex.

Residues involved in the construction of the nucleus in the form of amyloid, which in VL–VL complexes are included in the interface are—88, 101, and 102 and 32, 87, 91. Half of these residues build the interface in the discussed complexes. The situation is similar in the VL–VH domain complex.

## 3. Materials and Methods

### 3.1. Structural Codes of the Polypeptide Chain Form—Geometric Interpretation

The model of polypeptide chain structure description used in this work was presented in detail in [28,29,34]. For the purposes of this work only the basic elements are repeated. The use of Phi and Psi angles to determine the secondary structure is perfect from the point of view of determining the uniqueness of the conformation. This way of notating the structure of the polypeptide chain (except for the set of angles for the helical and β-structural form) is not able to suggest its geometric form. The notation of the polypeptide chain structure using two geometrical parameters—the radius of curvature (defined for a pentapeptide as a unit) (R) and the dihedral angle between adjacent planes of peptide bonds (V)—easily works on the imagination. It makes possible the prediction of the outline of the chain form also for structures at higher levels than secondary. The radius of curvature is a simple consequence of the dihedral (V) angle value between two successive peptide bond planes. The value of this angle is, in turn, a direct consequence of the Phi and Psi rotation.

The relationship between the value of angle V and the size of radius R (expressed on a logarithmic scale) takes the form of a parabola [29]. Values of the angle V close to 0 determine structures with a low radius value. This group includes all helical forms. Increasing the angle V results in increasing the radius of curvature reaching high values for V close to 180 deg. For the β-structural form, the angle V is nearly 180 deg and the radius of curvature reaches values striving for infinity (the extended form of the chain for certain sets of angle V takes the form of a straight line).

Values of the R radius are determined for the appropriate spatial orientation common to all conformations, for which the Z axis is determined by the average orientation of C=O bonds within peptide bonds. The radius of curvature is calculated for points that are projections of the positions of Cα atoms on the XY plane. The unit for which the V and R parameters are determined is a pentapeptide. This unit was adopted as the minimum for which it is possible to determine the secondary structure (helix, hair-pin turn, etc.).

The list of values of V angles and R radius marked on the Ramachandran map [29] illustrates the change in the geometric form covering the conformational space. Of course, the representation of the structure using the parameters V and R does not take into account chirality. That is why the map has a fully symmetrical form.

**Figure 7:**
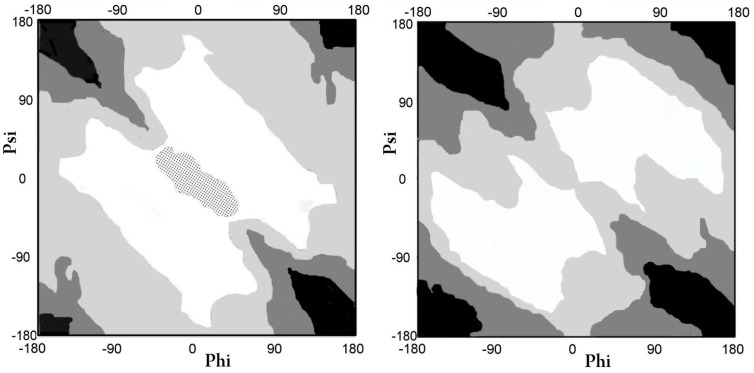
Map of Phi, Psi with marked value levels; (**A**) curvature radius; (**B**) the V angle; the gradation of color shows the increase in the value of the parameter. The area marked as black on map A—radius of curvature ln(R) > 5, black area for map B–V angle > 150 deg.; white area for map A—radius of curvature ln(R) < 2, white area for map B–V angle < 10 deg.; area covered with dots on map A—area not discussed.

Figure 7 shows the variability of the radius R (logarithmic scale introduced to avoid manipulating large values for the extended form) (Figure 7A) and the variation of the value of the angle V (Figure 7B).

**Figure 8:**
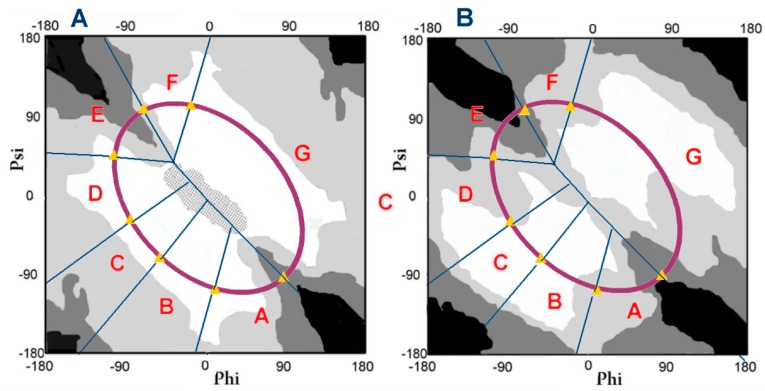
Distribution map; **A**—radius of curvature; **B**—the V angle; with a distinguished elliptical path expressing the optimal relationship between the radius of curvature and the V-angle. Zones resulting from the presence of local maxima of the probability of occurrence of the angles Phi and Psi are also marked.

The determined regression function for the relation ln (R) from V determines the optimal, relaxed structural form based on the geometric preference of Phi and Psi rotation. It produces the appropriate size of curvature in the effect. The identification of the location of conformational forms that implement this idealized relationship indicates an elliptical path connecting all areas representing forms with a specific secondary structure (Figure 8).

The structures obtained within this path can be described as being the result of preferences resulting only from the properties of the backbone. It comes from the assumption that this path is indicated by the optimal relationship between the orientation of the peptide bond planes and the resulting radius of curvature. This is why the Phi_e_ and Psi_e_ define the early stage of polypeptide deprived of the non-bonding interactions. They appear in the next steps of folding process. Inter-residual (inter-atomic) interactions result in increased packing. The consequence of which, is that the tertiary form is reached. The fuzzy oil drop model takes this into account. This model, apart from optimizing inter-atomic interactions, takes additionally into account the critical impact of the water environment on the folding process. It is expressed in the form of an external field directing the folding process towards the generation of a hydrophobic core [25,35,36,37]. The presence of a hydrophobic core is considered (next to disulfide bonds) as a factor determining the stability of tertiary structure.

By adopting the elliptical path as a conformational subspace for the early intermediate polypeptide chain, it becomes possible to perform a reverse operation—unfolding of the native structure—by changing the set of Phi and Psi angles present in the native structure to a set of Phi_e_ and Psi_e_ angles, where the subscript “e” means angles belonging to the limited conformational subspace expressed by elliptical path. The Phi_e_ and Psi_e_ angles determined on the criterion of the smallest distance between (Phi, Psi) may be used to classify the structure in respect to its original (early step) structural form. Additionally the areas (Figure 8) distinguished on the Ramachandran map may be used as structural cores identifying the conformation present in the native form.

Conducting such an operation for a non-redundant PDB structures set [26,38] reveals the presence of seven local maxima on the elliptical path [39]. These local maximum probabilities were treated as structural codes (codes A–G) for the early intermediate (Figure 8). A contingency table expressing the relationship between the tetrapeptide sequence and the corresponding (four) structural codes serves as a matrix for the design of the structure of the early intermediate is available on the internet [34]. In this work, it has been “re-calculated” on the basis of the non-redundant subset of the recent version of PDB resulting in some changes in the definition of structural codes which will be described later in this section. Details can be found in [40].

In the present work, however, the reference to this early intermediate model is limited only to determining the structural status of the polypeptide expressed precisely by means of the appropriate A–G structural codes.

It is also important to remind that the structural code C specifies the helix, E and F codes are β-structure codes, and the G code is the code for the left-handed helix. E and F codes differentiate structural forms traditionally classified as β-structural. However, it turns out that in the light of the classification we propose, this region shows diversity. The E code actually describes the Beta-structural form, while the F code refers to the terminal fragments of the β-structural sections and is present either at the initial position or at the end of the Beta-structural fragment [41]. This is illustrated by the maps of Phi and Psi angle distribution—e.g., lyase (1RKX [42]). The corresponding map is available at [38] by entering the ID 1RKX as ID [43,44].

Thus, the structure of a polypeptide can be written using A–G codes. The representation of individual zones on the Ramachandram map will be used to identify the diversity of structural forms of biologically active proteins and their amyloid forms.

### 3.2. Data

The subject of the analysis are two proteins whose biologically active and amyloid forms are available in the PDB database. These are the V domain of the immunoglobulin G light chain and Asynuclein, which is hereinafter referred to as ASyn. The list of analyzed proteins is given in Table 8.

The V domain is analyzed in its entirety; however, attention is focused mainly on sections 2–37 and 66–105 to enable comparative analysis of two different structural forms of this protein.

### 3.3. Calculation Procedure

The structure available in PDB is used to determine the angles Phi and Psi. In the next stage, structural codes are determined depending on the belonging of a given set of Phi and Psi to the appropriate fragment (local maximum) within the elliptical path and the corresponding area on the Ramachandran map.

The comparative analysis of the distribution of Phi and Psi angles for native and fibrillar forms consists in identifying positions where code differences occur. The location of these residues within the native and fibrillar structure allows to speculate on possible changes necessary for a given transformation and in particular the amyloid transformation.

### 3.4. Protein Status Analysis Taking into Account the Synergy

The geometric status of amino acids in a protein is confronted with its participation in building a form that is a kind of synergy. An expression of the role of a given amino acid in the construction of a cooperative system which is the structure of a protein is the status that a given residue has in relation to the structure of the hydrophobic nucleus. This term is related to the relation of the status of a given residue to the idealized distribution of hydrophobicity in the hydrophobic core, which is expressed mathematically using a 3D Gauss function defined on the protein body. The fuzzy oil drop model is a tool with which such assessment is possible [22,23,24,25]. Due to the repeated descriptions of this model, it is limited here only to remind one of the basic principles. For the description of the distribution of hydrophobicity within the protein molecule, a 3D Gauss function spread over a given molecule was adopted (size expressed by the parameters σ_x_, σ_y_, and σ_z_). The value of the function at any point in the protein body determines the idealized expected level of hydrophobicity (theoretical—Ti). This value is confronted with the level of hydrophobicity resulting from hydrophobic interactions with the neighbors of a given residue—referred to as Oi (observed). The comparison of these two values (after their normalization) allows to determine the status of a given residue in relation to the idealized state. It allows to determine the presence or absence of a hydrophobic core and possible local deformation of the O distribution versus the T distribution. The quantitative measure of such a deformation is determined by the divergence entropy function [45].

This model was used in this study to determine the role of individual residues, especially in relation to their participation in the structure or disruption of the hydrophobic nucleus structure. The parameters used to determine this status are RD (Relative Distance) measuring the relative distance of the O distribution to the T distribution and the R distribution (uniform, deprived of any form of hydrophobicity concentration, where each residue has a hydrophobicity status of 1 / N where N is the number of residues in the chain). RD values less than 0.5 indicate compliance of the actual and expected status. The use of this model allows to search for the causes of local incompatibility of the O distribution to the T distribution. The elimination of such residues indicates those residues that construct the hydrophobic nucleus according to the model.

This status is tracked in both native and fibrillar-amyloid structures of these proteins.

## 4. Conclusions

The combination of Figure 2; Figure 3 and of Figure 5; Figure 6 shows other system construction systems. It turns out that different close-ups of sections with high hydrophobicity result in a different final arrangement. The mechanism for generating individual structures is based—it seems—on different principles. Individual residues characterized by a specific set of features including hydrophobicity, in particular, seem to play different roles depending on the context—the environment in which they found themselves. While in the case of ASyn amyloid, the driver seems to be residues generating a hydrophobic nucleus in the form of a band micelle. In the case of amyloid obtained from the IgG V domain however, the influence of the external environment seems to be dominant. The environment—as can be speculated—directed the hydrophobic interaction in a different system than the V domain present in immunoglobulins.

The comparison of Phi and Psi angle changes with their geometric interpretation and the role in the construction of the factor stabilizing the tertiary structure seems to illustrate the differences in synergies present in the discussed systems.

The comparable analysis of amyloid form with the form appearing in physiological conditions (biologically active) is possible solely for two presented proteins due to the lack of experimentally recognized structures of other amyloids. However, the set of Aβ amyloids (different forms and different fragments of Aβ (1–42)) was the object of our comparable analysis. The reference structure for amyloid forms were the models generated by the top-programs oriented on protein structure prediction: I-TASSER and Robetta (CASP project - http://predictioncenter.org/). The results of these analyses for Aβ (1–42) and for tau-based-amyloid are available in [30,31] and [32], respectively.

For obvious reasons, the implication to the practical application in therapy is impossible due to large distance between the molecular level of our analysis and pharmacological research. However, any future practical application cannot be successful without the recognition of a molecular background of amyloidogenesis. The misfolding, together with the protein folding problem, may deliver some practical solutions in a near future.

## Figures and Tables

**Table 1:** Changes in location of structural codes. Rows in the table contain micelle-bound ASyn structural codes, columns—structural codes present in amyloid form (2N0A positions 30–100).

1XQ8 code → 2N0A code ↓	A	B	C	D	E	F	G	Σ
**A**	0	0	1	0	0	0	0	1
**B**	0	0	2	1	0	0	0	3
**C**	0	0	12	0	2	1	1	16
**D**	1	0	3	2	2	0	1	9
**E**	1	1	43	3	5	6	1	60
**F**	1	1	19	4	1	2	3	31
**G**	0	0	11	3	3	2	1	20
**Σ**	3	2	91	13	13	11	7	140

**Table 2:** Status (number) of residues involved in the construction of the hydrophobic core, showing the status in accordance with the fuzzy oil drop model And incompatible with the fuzzy oil drop model. The columns express the status in micelle-bound ASyn, the rows express the chain treated as a component of fibril.

ASyn → ASyn-FIBRIL ↓	Hydrophobic Core	Status Accordant	Status Not-accordant	Σ
**Hydrophobic Core**	4	2	19	25
**Accordant**	2	12	0	14
**Not-accordant**	5	15	12	32
**Σ**	11	29	31	71

**Table 3:** Status (number) of residues involved in the construction of the hydrophobic nucleus (Hydr core), showing the status in accordance with the fuzzy oil drop model (Acc.) and incompatible with the fuzzy oil drop model (Not accordant). The columns express the status in micelle-bound ASyn, rows—the string treated as an individual unit.

ASyn → Amyl Chain E ↓	Hydrophobic Core	Accordant	Not-accordant	Σ
**Hydr core**	11	2	15	28
**Accordant**	0	13	6	19
**Not-accordant**	0	13	11	24
**Σ**	11	28	32	71

**Table 4:** Status (number) of residues involved in the construction of the hydrophobic core, showing the status in accordance with the fuzzy oil drop model and incompatible with the fuzzy oil drop model. The columns express the status in chain E in as a fibril component, rows—status in an individual chain.

Chain E—in Fibril → Chain E—Individual ↓	Hydrophobic Core	Accordant	Not-accordant	Σ
**Hydr core**	16	3	9	28
**Accordant**	5	11	3	19
**Not-accordant**	4	0	20	24
**Σ**	25	14	32	71

**Table 5:** Changes in the location of structural codes. Rows—structural codes of the native form of the V domain, columns—structural codes present in the amyloid form. Numbers in brackets—number of residues of high intrinsic hydrophobicity difference in comparison with those present in amyloid form. A-G – zones on Ramachandran map distinguished according to local maxima on the ellipse path.

V domain → Fibril ↓	B	C	D	E	F	G	Σ
**A**			1				1
**B**			1	1	1		3
**C**	1			1	1	1	4
**D**							0
**E**	2	6 (1)	7 (3)	28 (3)	9 (2)	5	57 (9)
**F**				2	1	1	4
**G**						2	2
**Σ**	3	6 (1)	9 (3)	32 (3)	12 (2)	9	71 (9)

**Table 6:** The status of the V domain structural forms discussed in this work is expressed by the value of RD parameters determined for the respective fragments of single chains and as part of fibril.

Protein	Fragment	RD
**V domain**	2–111	0.573
	(2–30) + (66–105)	0.563
**Fibril**		
**Chains A–E**	(1–30) + (66–105)	0.773
**Chain C**	(1–30) + (66–105)	0.755

**Table 7:** Status (number) of residues involved in the construction of the hydrophobic core, showing the status in accordance with the fuzzy oil drop model and incompatible with the fuzzy oil drop model. The columns express the status in the V domain, the status in rows as it is observed in the fibrillar form. Only fragments 2–37 and 66–105 in domain V were analyzed. The numbers in parentheses give the number of residues with different levels of intrinsic hydrophobicity.

V domain → Fibril ↓	Hydrophobic Core	Accordant	Not-Accord	Σ
**Hydrophobic Core**	6 (1)	3	5 (2)	14 (3)
**Accordant**	7 (1)	16 (3)	1	24 (4)
**Not Accordant**	8 (1)	17 (1)	9	34 (2)
**Σ **	21 (3)	36 (4)	15 (2)	72 (9)

**Table 8:** A set of proteins being the subject of analysis along with their brief characteristics.

PROTEIN	PDB ID	Characteristics	Fragment	Reference
**ASyn**	1XQ8	Micelle-bound	30–100	[19]
	2N0A	Amyloid fibril	30–100	[18]
**V domain**	4BJL	Bence-Jones dimer	2–111 (2–37, 66–105)	[20]
	6HUD	Amyloid fibril	1–37, 66–105	[21]

## Abbreviations

ASyn	α-synuclein
